# A Mobile Phone-Based Program to Promote Healthy Behaviors Among Adults With Prediabetes Who Declined Participation in Free Diabetes Prevention Programs: Mixed-Methods Pilot Randomized Controlled Trial

**DOI:** 10.2196/11267

**Published:** 2019-01-09

**Authors:** Dina Griauzde, Jeffrey T Kullgren, Brad Liestenfeltz, Tahoora Ansari, Emily H Johnson, Allison Fedewa, Laura R Saslow, Caroline Richardson, Michele Heisler

**Affiliations:** 1 Veterans Affairs Center for Clinical Management Research Veterans Affairs Ann Arbor Healthcare System Ann Arbor, MI United States; 2 University of Michigan Medical School Ann Arbor, MI United States; 3 Institute for Healthcare Policy and Innovation University of Michigan Ann Arbor, MI United States; 4 University of Michigan School of Dentistry Ann Arbor, MI United States; 5 University of Michigan School of Nursing Ann Arbor, MI United States; 6 University of Michigan School of Public Health Ann Arbor, MI United States

**Keywords:** autonomous motivation, behavioral change, mHealth, mobile phone, prediabetes, prevention, type 2 diabetes mellitus

## Abstract

**Background:**

Despite evidence that Diabetes Prevention Programs (DPPs) can delay or prevent progression to type 2 diabetes mellitus (T2DM), few individuals with prediabetes enroll in offered programs. This may be in part because many individuals with prediabetes have low levels of autonomous motivation (ie, motivation that arises from internal sources) to prevent T2DM.

**Objective:**

This study aims to examine the feasibility and acceptability of a mobile health (mHealth) intervention designed to increase autonomous motivation and healthy behaviors among adults with prediabetes who previously declined participation free DPPs. In addition, the study aims to examine changes in autonomous motivation among adults offered 2 versions of the mHealth program compared with an information-only control group.

**Methods:**

In this 12-week, parallel, 3-arm, mixed-methods pilot randomized controlled trial, participants were randomized to (1) a group that received information about prediabetes and strategies to prevent T2DM (control); (2) a group that received a mHealth app that aims to increase autonomous motivation among users (app-only); or (3) a group that received the app plus a physical activity tracker and wireless-enabled digital scale for self-monitoring (app-plus). Primary outcome measures included rates of intervention uptake (number of individuals enrolled/number of individuals assessed for eligibility), retention (number of 12-week survey completers/number of participants), and adherence (number of device-usage days). The secondary outcome measure was change in autonomous motivation (measured using the Treatment Self-Regulation Questionnaire), which was examined using difference-in-difference analysis. Furthermore, we conducted postintervention qualitative interviews with participants.

**Results:**

Overall, 28% (69/244) of eligible individuals were randomized; of these, 80% (55/69) completed the 12-week survey. Retention rates were significantly higher among app-plus participants than participants in the other 2 study arms combined (*P*=.004, χ^2^). No significant differences were observed in adherence rates between app-only and app-plus participants (43 days vs 37 days; *P*=.34). Among all participants, mean autonomous motivation measures were relatively high at baseline (6.0 of 7.0 scale), with no statistically significant within- or between-group differences in follow-up scores. In qualitative interviews (n=15), participants identified reasons that they enjoyed using the app (eg, encouraged self-reflection), reasons that they did not enjoy using the app (eg, did not consider personal circumstances), and strategies to improve the intervention (eg, increased interpersonal contact).

**Conclusions:**

Among individuals with prediabetes who did not engage in free DPPs, this mHealth intervention was feasible and acceptable. Future work should (1) examine the effectiveness of a refined intervention on clinically relevant outcomes (eg, weight loss) among a larger population of DPP nonenrollees with low baseline autonomous motivation and (2) identify other factors associated with DPP nonenrollment, which may serve as additional potential targets for interventions.

**Trial Registration:**

ClinicalTrials.gov NCT03025607; https://clinicaltrials.gov/ct2/show/NCT03025607 (Archived by WebCite at http://www.webcitation.org/73cvaSAie)

## Introduction

Type 2 diabetes mellitus (T2DM) is a key driver of death, disability, and health care spending in the United States [1,2]. In 2015, >30 million US adults had T2DM, while 84 million more were estimated to have prediabetes, a condition associated with an increased risk of developing T2DM [1]. Diabetes Prevention Programs (DPPs) can help individuals with prediabetes to achieve modest weight loss through diet and physical activity changes that reduce the 3-year risk of developing T2DM by >50% [3,4]. Accordingly, DPPs are now offered throughout the United States, and a growing number of health plans [5], including Medicare [6], offer DPPs to eligible plan members at no out-of-pocket cost.

Despite the widespread availability of DPPs and public health efforts that aim to increase DPP engagement, rates of program uptake remain extremely low [7,8]. To date, strategies to increase the DPP uptake have targeted extrinsic barriers to participation (eg, lack of time and cost) through the provision of Web-based DPPs [9] and insurance coverage with limited success [5,6]. In contrast, to our knowledge, no current strategies address intrinsic barriers to participation, such as low levels of motivation to prevent T2DM, yet prior literature suggests that a lack of motivation may be a key barrier to DPP engagement [10]. Accordingly, it is necessary to develop and test scalable approaches to help increase the motivation of millions of Americans who have prediabetes but are not yet taking actions to reduce their risk of progression to T2DM. Such strategies may be most effective if they draw on the principles of self-determination theory to increase autonomous motivation (ie, motivation that arises from internal sources and aligns with personal interests and values) [11,12]. Greater levels of autonomous motivation correlate positively with dietary adherence [13], weight loss [14,15], physical activity [16,17], DPP participation [10], and maintenance of healthy behaviors over time [18,19].

Mobile health (mHealth) apps that are easy to use and do not require a significant time commitment may be effective and highly scalable approaches to increase autonomous motivation to prevent T2DM among those with prediabetes [20,21]. One mHealth app under development, for example, promotes personal well-being by helping users to (1) identify their core values (eg, to be a good parent); (2) reflect on their adherence to these values; and (3) develop the energy and willpower to live in accordance with their core values by improving key health behaviors (eg, sleep, physical activity, and diet). The mHealth app integrates user-entered health information with contextual data (eg, local weather and day of the week) and then delivers brief tailored messages and health tips to help individuals gain awareness of and control over the factors in real-time that influence their ability to engage in self-care behaviors. In this way, the app helps users connect their daily habits and routines with personal interests and values, thereby strengthening autonomous motivation to engage in healthy behaviors. Yet, it is not known whether adults who have already declined participation in offered DPPs are willing to participate in and then engage in offered mHealth programs.

Accordingly, in this 3-arm, mixed-methods pilot randomized controlled trial, we tested the feasibility of recruiting DPP nonenrollees into an mHealth intervention and the acceptability of the mhealth program—used alone and also in conjunction with Fitbit devices (eg, activity tracker and wireless internet-enabled scale) to encourage self-monitoring—among individuals with prediabetes who had declined participation in Web-based or face-to-face DPPs offered at no out-of-pocket expense by their health plans. As we hypothesized that autonomous motivation would be a key proximal mediator of behavioral changes among those who did engage with the intervention, we also estimated the change in study participants’ autonomous motivation during the 12-week intervention period. In addition, as Fitbit devices can enhance motivation and self-efficacy through self-determination theory principles [11,22] and self-monitoring techniques [23], we further hypothesized that autonomous motivation to prevent T2DM would increase to a greater degree among individuals who used the app in conjunction with Fitbit devices compared with individuals who used the app alone or who were assigned to the control arm.

## Methods

### Design

**Figure 1:**
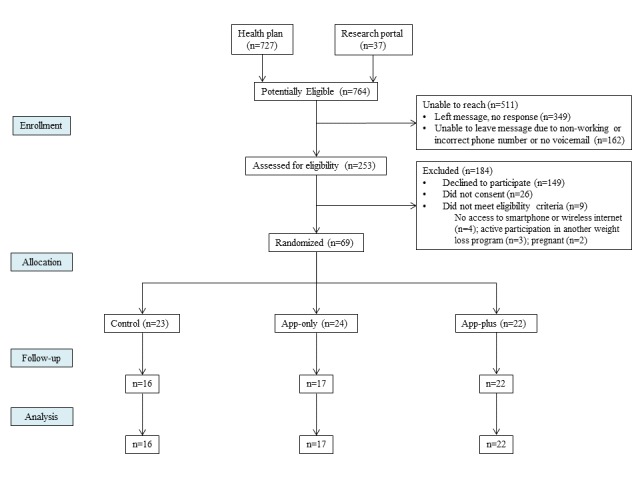
Study flow diagram.

We conducted a 12-week, parallel, 3-arm, mixed-methods pilot randomized controlled trial between May 2017 and February 2018 (NCT03025607). Overall, 69 participants were randomized to 1 of 3 arms (Figure 1) as follows: (1) a group that received information about prediabetes and evidence-based ways to decrease the progression to T2DM, as well as a list of resources for mHealth tools for monitoring diet, physical activity, and weight (control group); (2) a group that received the same information as the control group and the mobile smartphone app (app-only); and (3) a group that received the same information as the control group, as well as the mobile smartphone app and Fitbit devices (eg, activity tracker and wireless internet-enabled scale) whose results were automatically synced with the mobile app and informed the app’s tailored messaging (app-plus). This commercially available app is hosted on Amazon Web Services, with all data encrypted at rest, in transit, and when backed up. We used a mixed-methods sequential explanatory design [24]; quantitative and qualitative data were collected in 2 consecutive phases during the study and then integrated into the final stage of data analysis. This approach enabled us to interpret our quantitative data in the context of qualitative participant experiences. The protocol was approved by the University of Michigan Institutional Review Board (HUM00111389).

### Setting and Participants

The intervention was delivered remotely. The inclusion criteria were as follows: (1) nonenrollment in a DPP at least 6 months after invitation from one’s health plan to participate at no out-of-pocket cost (ie, DPP nonenrollee); (2) prediabetes based on American Diabetes Association criteria of a hemoglobin A_1c_ (HbA_1c_) level between 5.7% and 6.4%; (3) access to a personal smartphone; and (4) access to home wireless internet. We excluded women who were pregnant or intended to become pregnant during the intervention period.

We had a unique opportunity to recruit locally, as our institution’s self-funded health insurers recently began to offer face-to-face and Web-based DPP options to health plan members (ie, employees, retirees, and students of the University of Michigan or their dependents) with prediabetes at no out-of-pocket cost, yet only 6% of program invitees enrolled in a DPP within the first 6 months (September 2015-February 2016) of the program (unpublished communication). For this pilot study, the University’s health plans provided the study team with a random 18.5% (727/3926) sample of DPP nonenrollees. In addition, we posted study recruitment information on the University’s health research website to allow interested and potentially eligible individuals to contact our team directly [25]. We attempted to contact all individuals by telephone to invite them to participate in this study. Three attempts were made to contact each individual; a voicemail with the study team’s contact information was left after the second attempt. Individuals interested in study participation were screened by telephone to ensure they met the study eligibility criteria, and informed consent was obtained electronically using the RedCap survey platform [26].

### Allocation

Individuals who met the study inclusion criteria, provided written informed consent, and completed a baseline questionnaire were assigned to the 3 study groups using 1:1:1 central computerized randomization. The allocation sequence was generated using Stata 14. A Web-based tool, the University of Michigan computerized randomization system (Treatment Assignment Tool-UM, TATUM), was used to allow for blinded treatment allocation. We used stratified randomization with variable block lengths to ensure a balance of age and gender between groups. Owing to the nature of the intervention, it was not possible to blind participants; those performing the analyses, however, were blinded to treatment assignment arms.

### Intervention

All participants received the Center for Disease Control and Prevention’s 2-page educational handout on prediabetes and evidence-based strategies to prevent the progression to T2DM, as well as a list of free mHealth resources for monitoring weight and physical activity. In addition, app-only and app-plus participants received emailed instructions for setting up the app. App-plus participants received their Fitbit devices and set-up instructions through postal mail. A study team member was available by telephone and email to answer study-related questions and troubleshoot technical issues. Three app-only participants (12.5%) contacted the study team to request assistance with the app set-up. Six app-plus participants (27.2%) contacted the study team to request assistance with setting up the app or Fitbit devices. Once participants began using the app and/or Fitbit devices, there was no further contact with the study team for technology support, and there was no additional planned contact between participants and study team members during the study period.

App-only and app-plus participants were asked to use the smartphone app daily to chart the following health-related habits and behaviors: (1) Sleep; (2) Presence; (3) Activity; (4) Creativity; and (5) Eating (S.P.A.C.E). In addition to charting S.P.A.C.E. on a daily basis, users were asked to reflect on and chart their alignment with personal core values (ie, life purpose); these charted data then informed tailored messages and health tips, as well as predictions of an individual’s energy and willpower for the coming day. These predictions are intended to help individuals gain awareness of and control over the factors that influence their health behaviors. Furthermore, app-plus participants were asked to use the Fitbit scale and activity tracker daily to self-monitor weight and physical activity, respectively. These devices interfaced with the app platform such that the Fitbit data informed delivered tailored messages and health tips.

Within the app, users were asked if they wished to receive a daily reminder to chart their day. Users who desired a daily reminder received a push notification at a self-selected time, which reminded them to chart their day. Users who did not desire a daily reminder received no other reminders to use the study-specific device(s).

### Primary Quantitative Measures: Feasibility and Acceptability

We evaluated the intervention’s feasibility (uptake and retention rates) and acceptability (adherence and qualitative experience). The program feasibility was determined by calculating the intervention uptake rate, defined as the number of participants recruited to the intervention divided by the total number of potentially eligible participants. Furthermore, we calculated the rate of intervention uptake among only those who were reached by telephone. To determine the study retention rate, we calculated the rate of completion of the 12-week survey among all individuals enrolled in the study.

Among app-only and app-plus participants, we measured adherence to the app, defined as the number of days that users entered data into the app during the 12-week intervention period. Among app-plus participants, we measured participant adherence to the Fitbit activity tracker and scale, defined as the number of total days that each of these devices were used during the intervention period.

### Secondary Quantitative Measures: Web-based Surveys

Prior to randomization, individuals who consented to study participation were asked to complete a Web-based survey via RedCap, a secure Web app [26]; this first survey was used to collect demographic and socioeconomic information, including age, gender, race, ethnicity, education, and household income. We used the 7-item, validated Treatment Self-Regulation Questionnaire (TSRQ) to measure autonomous motivation to prevent T2DM [27]. Following the 12-week intervention period, participants were emailed a link to the second survey. This survey asked participants to complete the same validated instrument that was collected at baseline. Participants were provided with a US $10 gift card following the completion of each survey (ie, baseline and 12-weeks).

### Qualitative Measures: Semistructured Telephone Interviews

Following the 12-week intervention period, we invited all individuals in the app-only and app-plus groups to participate in a semistructured telephone interview. We planned to conduct a minimum of 20 interviews with additional interviews to be conducted only if thematic saturation was not achieved at this point [28]. During the interviews, we explored participants’ experiences with the app and Fitbit devices, if applicable. In addition, participants discussed health behavioral changes that occurred as a result of program participation and suggested potential strategies to strengthen and refine the intervention. Of note, interview participants received a US $20 gift card as compensation for their time.

### Sample Size

Based on prior studies of autonomous motivation among University of Michigan employees [10], we anticipated that the baseline level of autonomous motivation to prevent T2DM among those who declined DPP participation after invitation by their health plan to be 5.7 (measured on a 1-7 scale with 1 being the lowest and 7 being the highest). During the 12-week intervention period, we anticipated that autonomous motivation would increase by 0.6 points in the app-only arm and by 0.8 points in the app-plus arm. Assuming an SD of 1.0 for change in autonomous motivation in both arms, we required 29 participants in each arm to provide 80% power to detect these changes in autonomous motivation in the intervention arms compared with the control arm. Prior research demonstrates that a 0.5-point increase in autonomous motivation is associated with markedly higher weight loss and increased physical activity compared with individuals who did not achieve this increase in autonomous motivation [18]. To account for the possibility that some participants may be lost to follow-up during our 12-week intervention, we conservatively inflated our sample size by 20% to enroll 35 participants in each arm.

Owing to administrative changes within the health plan and competing research interests within our institution, the plan provided us with a limited list (727/3926, 18.5% sample) of individuals who were potentially eligible for our study. As such, we were unable to meet our recruitment target. Using our realized sample size (n=69), we conducted a post-hoc power analysis, which showed that we had 80% power to detect a mean difference of ≥0.38 in the intervention arms compared with the control arm.

### Statistical Analysis

#### Quantitative Data Analysis

We used logistic regression to compare differences in rates of engagement between the 2 intervention arms. We used linear regression to compare differences in adherence (ie, app-usage days) between the intervention arms. In addition, we compared changes in autonomous motivation among app-only and app-plus participants versus control participants using a difference-in-differences analytic approach. For continuous outcome measures, we modeled the effect using linear regression, and for dichotomous outcomes, we modeled the effect using logistic regression. The difference-in-difference is an interaction term between a categorical variable indicating the study group (ie, control vs app-only vs app-plus) and a categorical variable indicating the data collection time-point (ie, baseline vs 12-week follow-up). The difference-in- differences design accounts for the possibility that temporal trends unrelated to the intervention may have influenced the study outcome. All analyses were conducted using Stata 14 (StataCorp LP).

#### Qualitative Data Analysis

Semistructured interviews were recorded, transcribed verbatim, and imported into qualitative analysis software, Dedoose (SocioCultural Research Consultants, Los Angeles, CA, USA). Two investigators independently read and coded transcribed interviews. Interviews were then coded jointly using consensus conferences and analyzed using directed content analysis [29]. Although we planned to conduct a minimum of 20 interviews, no new themes emerged after coding 8 transcripts. Given that thematic saturation was achieved earlier than anticipated, we conducted only 15 interviews.

## Results

### Intervention Uptake

Figure 1 shows the flow of participants through the study. Contact information for a total of 740 individuals identified as potentially eligible for study participation was provided to us by their health plan, and 37 individuals identified as potentially eligible by self-report through a health research portal. We were unable to reach the majority of potentially eligible individuals (527/777, 68%). Among 253 individuals assessed for eligibility, 244 were eligible to participate, and 28% (69/244) of these eligible individuals consented to study participation and were randomized to 1 of 3 study arms.

### Baseline Characteristics

Demographic and socioeconomic characteristics were assessed at baseline (Table 1). Most participants were females (64%), white (65%), and educated, with 91% attaining education beyond high school. The mean age was 51.7 years (11.2). At baseline, mean autonomous motivation score was 6.0 (SD 1.0) among control group participants, 5.8 (SD 1.0) among app-only participants, and 6.0 (SD 1.0) among app-plus participants.

### Quantitative Analyses

#### Retention

Among those randomized (n=69), 55 (80%) completed the 12-week survey. Rates of survey completion varied across study arms. Among participants in control, app-only, and app-plus groups, completion rates were 70% (16/23), 71% (17/24), and 100% (22/22), respectively. Retention differed significantly between app-plus participants and participants in the other 2 study arms combined (*P*=.004, *χ*^2^).

#### Adherence

During the 12-week (84-day) intervention period, app-only participants used the app for a mean of 43 days (SD 26.6; 51% of study days), while app-plus participants used the app for a mean of 37 days (SD 26.2; 44% of study days); *P* value (.34).

Among app-plus participants (n=22), 73% (16/22) used the Fitbit activity tracker for a mean of 32 days (SD 12.0), and 59% (13/22) used the Fitbit scale for a mean of 15.9 days (SD 15.4). Of note, 3 app-only participants paired their personal Fitbits with the app, although they were not instructed to do so as part of the study; these individuals used the Fitbit for a mean of 21 (SD 8) days.

#### Exploratory Quantitative Outcomes

Table 2 shows the changes in autonomous motivation scores across the study groups. The scores were measured on a scale of 1-7 using the TSRQ; higher scores indicate greater levels. No statistically significant within- or between-group differences were observed in self-reported autonomous motivation.

**Table 1:** Baseline characteristics of study participants.

Characteristics		Control (n=23)	App-only (n=24)	App-plus (n=22)
**Demographics**				
	Mean age (years), mean (SD)	51.3 (11.0)	52.1 (12.0)	51.6 (11.1)
	Female, n (%)	15 (65.2)	15 (62.5)	14 (63.6)
	Body mass index in kg/m^2^, mean (SD)	33.0 (10.4)	30.7 (9.3)	33.4 (7.8)
	Minority race^a^, n (%)	6 (28.6)	11 (45.8)	7 (31.8)
**Education, n (%)**				
	High school graduate	3 (13.0)	1 (4.2)	1 (4.6)
	More than high school	20 (87.0)	22 (91.7)	21 (95.5)
**Household income (in US $), n (%)**				
	<50,000	7 (31.8)	6 (27.3)	6 (28.6)
	50,000-100,000	8 (36.4)	12 (54.6)	6 (28.6)
	>100,000	7 (31.8)	4 (18.2)	9 (42.9)
Autonomous motivation to prevent type 2 diabetes mellitus^b^, mean (SD)		6.01 (1.0)	5.80 (1.0)	5.96 (1.0)

^a^Defined as any race other than white.

^b^Measured on a scale of 1-7 using the Treatment Self-Regulation Questionnaire. Higher scores indicate greater levels.

**Table 2:** Difference-in-difference analysis for autonomous motivation scores at 12 weeks compared with baseline.

Study groups	Baseline mean (SE)^a^	12-week mean (SE)	*P* value	
			Within-group difference at 12 weeks)	Difference-in-difference from baseline to 12 weeks
Control (n=16)	6.01 (0.21)	5.87 (0.25)	–0.14 (.57)	Not applicable
App-only (n=17)	5.80 (0.21)	5.88 (0.25)	0.08 (.73)	0.22 (.51)
App-plus (n=22)	5.96 (0.21)	5.90 (0.21)	–0.06 (.72)	0.08 (.77)

^a^All values in this table are predicted from the model.

#### Participant Experiences With the Intervention

Among 24 app-only participants invited to participate in an interview, 5 individuals (20%) agreed to take part. Among 22 app-plus participants invited to participate in an interview, 10 individuals (45%) agreed to take part. During these interviews, key themes emerged regarding participants’ perceptions of the app, capturing those aspects of the app that they liked or disliked (Table 3).

Among 13 interviewees who identified components of the app that they enjoyed, the majority (n=8) appreciated the app’s support for self-reflection. For example, one app-only participant commented, “I liked how you had to rank how [you were] feeling [each day]...I thought [that was] an interesting way just to take a step back, just sort of a self-assessment.” Others (n=5) noted that the app supported adherence to healthy behaviors over time through daily charting of health habits (eg, diet, physical activity, and sleep), light-touch health tips, and educational videos. As noted by one app-only participant, “[the app] was a good reminder...to help push [me] to keep moving...doing more and more. ”

Among 11 participants who identified components of the app that they did not enjoy, almost half (n=5) commented that daily use of the app felt burdensome as a result of the minimal day-to-day variation in individual health behaviors, redundancy of educational content, and perceived arbitrariness of future predictions. One app-plus participant commented that he was initially motivated to chart daily; however, he also said:

...after a while...I lost interest in trying to understand what it was doing for me other than just keeping track and telling me that tomorrow it's supposed to rain. You might have a bad day.

**Table 3:** Participants’ perceptions of the mHealth app and representative quotes.

Participant perceptions	Representative quotes
Encouraged reflection on factors that influence health	“[The App] helps me think about how I can use [my family] to support me...even though they live far away, I can just have a conversation with them and try to use them as part of my support, as well as my community, which are my friends, my church, my school parents, things like that. ’Cause I realize that these are actually part of the environment that could help me be a healthier person.” *(App-only)* “It makes me decompress from my day and just think, “How could I have made my day better? What did I do? What didn’t I do?” *(App-plus)*
Supported healthy behaviors	“I was more conscious of what I ate. I started...drinking more water, less caffeinated beverages, less carbonated beverages...I wasn’t as tired. I set a goal where I was going to bed by a certain time.” *(App-only)* “When I go see my doctor, it’s kind of like, ‘...you need to exercise more...you need to change your diet’. But the nice thing about [the app] was [it] broke it down into these things that you could learn about that allowed you to have a better understanding [of] your health condition...and also how you can sort of prevent certain health risks from happening.” *(App-only)*
Daily use was burdensome	“There [were] a lot of questions about how I feel today...it just seemed to be a little bit of the same old same old every day or every time I looked at it.” *(App-only)* “[The App] just got too time consuming and I just lost interest in keeping track of all that data. It just became too overwhelming, I was doing other things.” *(App-plus)*
Failed to consider personal circumstances	“I [have] paroxysmal afib, which means some days...I didn’t feel very energetic...[But] there was no way to [tell the app], ‘this day is different for completely non-purpose related reasons’.” *(App-plus)* “Sometimes [things] go completely awry and just change what’s gonna happen, my plan for the day. So outside factors...absolutely [have] an impact on your day. So you can still be positive, you can still have a plan for exercise. But sometimes, there’s things that come up...” *(App-plus)*

Four individuals voiced frustration with certain health tips delivered by the app, as these failed to recognize personal or environmental circumstances that transiently influenced one’s health habits, energy, or willpower. For example, an app-plus participant noted:

...time I was on vacation, and I have to work really hard to get the vacation. And I had a drink every single day, not a lot, just maybe one, and there was a thing that came up about sleeping and limiting your alcohol intake, and I'm going, “Oh, for God's sakes. I shouldn't even put any of that down.”

Among app-plus interviewees, all (n=10) used the Fitbit activity tracker, and most (n=6) noted that it facilitated engagement in routine physical activity. For example, one participant said:

I live about two miles away from our office. I ended up much more in the mode of, “I'm gonna walk if it's all possible.”

Several participants specifically appreciated the activity tracker’s concrete step count goal, and one noted:

...looking at [activity] from a more lucid mathematical standpoint was very helpful. It made me more active without having to engage in an abrupt behavior or thought change.

Among app-plus interviewees, 8 used the scale and appreciated the ease with which the data synced with the Fitbit app. One participant commented “I thought it was wonderful...[you just] step on this little device and magically it goes into your statistics, and I get a running account of if my weight's going up or down or whatever.” Similarly, another noted, “I just step on the scale and it's recorded in the Fitbit app, and that was handy ’cause it keeps a record.”

Thirteen interview participants identified specific health behavioral changes that resulted from participation in this intervention. These included increased physical activity (n=9), improved dietary habits (n=8), increased awareness of other factors that influence health and well-being such as social connectedness and adequate sleep (n=6).

Thirteen interview participants suggested strategies to enhance the intervention. Five participants recommended adding some level of “human contact” to support behavioral change better. An app-plus participant commented, “I would have enjoyed talking with an actual person…to get more advice.” Three participants thought that more concrete goal-setting could better help participants achieve health goals. For example, an app-plus participant noted:

[The app] didn’t seem to offer...concrete things to do. It just sort of asking me to reflect on how I did in sort of pretty unstructured ways. [I wanted to] be able to set concrete things to do...Instead of just asking me how active I was, ask if I [met my goal of] walking at least four miles a day...

Another suggested the addition of concrete nutritional advice so that participants may know:

...what not to eat, what to eat, and what are the nutritional values of different things, and how you can manage your day based on your work schedule, when you should be eating, what you should be eating, how much you should be eating and you could still feel hungry.

## Discussion

### Principal Findings

To the best of our knowledge, this is the first study to test an intervention to support healthy behaviors among individuals with prediabetes who had recently declined participation in Web-based or face-to-face DPPs offered at no cost. Our findings demonstrate that it is indeed feasible to recruit DPP nonenrollees to an mHealth intervention. Nearly one-third of eligible individuals enrolled in this intervention despite previously declining to participate in free Web-based and group-based DPPs offered by our University’s self-funded insurers. Furthermore, the app—used alone and also in conjunction with Fitbit devices—was acceptable among intervention group participants, as indicated by high levels of adherence and positive qualitative experiences.

Retention differed markedly between app-plus participants and participants in the other 2 study arms. One explanation for between-arm differences in retention is that the Fitbit devices enhanced the intervention’s acceptability and perceived value to participants. Fitbit devices incorporate established behavioral change techniques (eg, self-monitoring, feedback, and goal-setting) [30], and our qualitative data suggest that participants’ enjoyment of these features may have motivated study retention. Alternatively, because app-plus participants received a more robust intervention, they may have felt a greater sense of obligation to the study, making them more likely to complete the 12-week survey. Adherence to the app did not differ markedly between intervention groups. In qualitative interviews, participants indicated that they discontinued the app daily use owing to the perceived burden of data entry and lack of personal relevance. These reasons for the discontinued app use are consistent with those previously described in the literature [31].

We examined the intervention’s preliminary efficacy on autonomous motivation to prevent T2DM, which we hypothesized to be a key proximal mediator of behavioral change. Our analyses did not demonstrate statistically significant differences in levels of autonomous motivation between intervention arms. It is plausible that we were unable to discern changes in autonomous motivation owing to higher-than-predicted baseline levels of autonomous motivation and resultant ceiling effect of the TSRQ. While high baseline levels of autonomous motivation may have occurred by random chance, it is also possible that these high levels identify a nonrandom subset of DPP nonenrollees who are motivated to prevent T2DM, yet face other barriers to DPP enrollment (eg, lack of time). Accordingly, high levels of postintervention autonomous motivation across arms may reflect intrinsic characteristics of our study participants rather than the intervention’s effect. Given the importance of autonomous motivation for initiating and sustaining healthy behaviors, it is critically important to characterize autonomous motivation levels among the broader population of DPP nonenrollees and conduct a larger-scale effectiveness trial to examine changes in autonomous motivation specifically among individuals with lower baseline levels. Another possibility is that 12 weeks was a too short period to observe marked improvements in autonomous motivation; prior studies have examined changes over longer time periods. In addition, future research should explore factors other than low levels of autonomous motivation that may deter the DPP uptake to inform additional targeted interventions to address these barriers specifically.

Mobile smartphone apps and other mHealth technologies are increasingly used as tools to promote lifestyle changes [32], and technology-assisted translations of the DPP have been used to improve program reach [33]. While such programs may be cost-effective and convenient, their effectiveness is variable, and little is known about the populations most likely to engage in or benefit from mHealth programs [33,34]. Without such knowledge, these programs cannot be adequately tailored or disseminated to those most likely to benefit from them. In this study, we specifically recruited individuals who declined participation in free DPPs, and, through qualitative interviews, we gained insight into key opportunities to augment the effectiveness of this low-intensity mHealth program. Notably, several participants expressed a desire for enhanced interpersonal contact during the study period. In addition to fostering a sense of personal connection, such contact may facilitate concrete goal-setting and follow-up, thereby optimizing behavioral change outcomes; prior mHealth interventions for weight loss, for example, have proven most effective when combined with health coaching [35-37]. Furthermore, we demonstrated that some interpersonal contact is necessary for program on-boarding.

### Limitations

First, we aimed to enroll 35 individuals in each study arm, but we were unable to meet this recruitment target owing to administrative changes within the health plan and competing research interests within our institution. Thus, we were not powered to detect our hypothesized changes in autonomous motivation, and baseline autonomous motivation scores were higher than expected among our study participants. Second, we recruited individuals from a single regional health plan, and our results may not be generalizable to other populations; our study participants were highly educated with access to personal smartphones and home wireless internet. As such, they may have been more willing and able to engage in a mHealth intervention for diabetes prevention than less educated or resourced individuals [38,39]. Future work could aim to engage a broader cohort of DPP nonparticipants with lower levels of baseline autonomous motivation and more diverse sociodemographic characteristics. Finally, because this was a pilot study designed to assess the feasibility and acceptability, we were not powered to examine changes in clinically relevant behaviors for T2DM prevention (eg, weight loss and increased physical activity); these outcomes warrant investigation in larger-scale trials.

### Conclusions

National initiative [40,41] and policies [42] promote DPPs as the dominant diabetes prevention strategy, yet the ability of DPPs to improve population health is compromised by the low program uptake. Alternative strategies are urgently needed to help the large majority of individuals with prediabetes prevent T2DM and T2DM-related complications. In this pilot study, we demonstrate the feasibility and acceptability of a low-intensity mHealth program among some individuals with prediabetes who do not desire participation in formal DPPs. However, additional strategies are also needed to engage those DPP nonparticipants who also decline mHealth programs. In future work, we will refine the existing intervention by incorporating participant-identified preferences for increased interpersonal contact and concrete goal-setting. We will then conduct a larger-scale effectiveness trial to examine changes in key proximal mediators of behavioral change (eg, autonomous motivation and self-efficacy), as well as changes in clinically relevant outcomes (eg, weight, HbA_1c_, and physical activity). Furthermore, we will explore needs and preferences for lifestyle change approaches among a broad population of DPP nonparticipants, and these data will be used to develop additional tailored interventions for T2DM prevention.

## Appendix

Multimedia Appendix 1 CONSORT‐EHEALTH checklist (V 1.6.1).

## Abbreviations

DPP: Diabetes Prevention Program
HbA _1c_: hemoglobin A_1c_
mHealth: mobile health
T2DM: type 2 diabetes mellitus
TSRQ: Treatment Self-Regulation Questionnaire
